# Electrochemical proton-coupled electron transfer of an anthracene-based azo dye†

**DOI:** 10.1039/d0ra01643h

**Published:** 2020-04-14

**Authors:** Amanda N. Oldacre, Elizabeth R. Young

**Affiliations:** Department of Chemistry, St. Lawrence University Canton New York 13617 USA ery317@lehigh.edu +1-610-758-6536; Department of Chemistry, Lehigh University Bethlehem Pennsylvania 18015 USA

## Abstract

Herein, we report the thermodynamics, kinetics, and mechanism for electrochemical proton-coupled electron transfer involving the anthracene-based azo dye azo-OMe. The peak reduction potential of azo-OMe with organic acids spanning the p*K*_a_ range of 2.6–23.51 shows a dependence upon the p*K*_a_ of the acid when the acid p*K*_a_ falls between 8 and 20 (in acetonitrile). A potential-p*K*_a_ diagram is constructed and used to estimate the p*K*_a_ of the azo-OMe species. Heterogeneous electron-transfer rate constants are obtained using rotating disk electrode voltammetry in combination with Koutecký–Levich and Tafel analysis. Electrochemical analysis shows that the reactions are diffusion limited and are kinetically controlled by the electron-transfer step. Kinetic isotope studies indicate a concerted proton, electron transfer event occurs in the p*K*_a_-dependent range when using trifluoroacetic acid.

## Abstract 2

Herein, we report the thermodynamics, kinetics, and mechanism for electrochemical proton-coupled electron transfer involving the anthracene-based azo dye azo-OMe.

## Graphical abstract

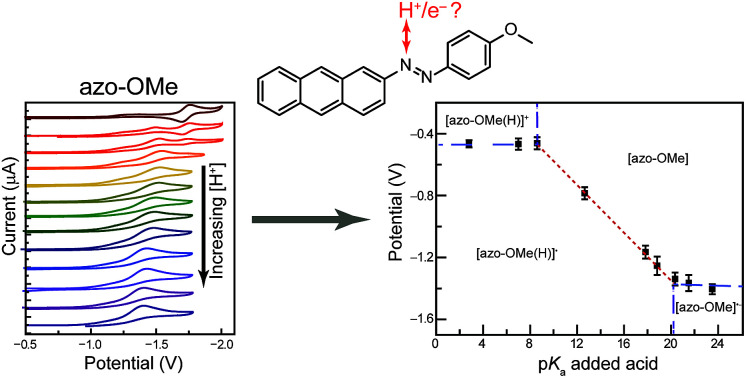

## Introduction

Nature has evolved a mechanistic advantage in carrying out electron-transfer steps, as well as associated chemical reactions, by coupling the movement of electrons along reaction pathways to proton motions. The coupling of proton and electron movements play an important role in energy conversion reactions, energy storage, and small molecule activation reactions.^1–5^ Detailed mechanistic understanding of charge transfer and proton-coupled electron transfer (PCET) can enable a sustainable carbon-neutral energy economy. Fundamental mechanistic studies, wherein the PCET mechanism can be controlled, are of vast importance for energy conversion reactions.

Over the past decade, PCET at electrochemical interfaces has gained attention due to the significant role electrochemical PCET plays in energy conversion reactions and catalysis.^6–15^ Experimental, theoretical, and computational work has shown non-innocence of protons at electrochemical interfaces.^14–25^ Indeed, many chemical reactions, including reactions involving homogeneous catalysis and heterogeneous catalysis at electrode surfaces show a clear dependence on the pH of the solution.^15^

Protons influence the Fermi level at the electrochemical interface thereby controlling the thermodynamics and, as a result, the kinetics of electrochemical PCET reactions. Transition state theory makes this connection between activation energy and reaction kinetics. The activation energy of stepwise PCET compared to the activation energy of concerted PCET dictates the observed mechanism. For example, if the activation energy of the concerted PCET reaction is less than the activation energy of the sum of the individual proton- and electron-transfer steps, then the concerted reaction will be kinetically favoured. It follows, then that the dependence of the reactions on pH results from a decoupling of the electron and proton transfer steps in PCET reactions caused by one step of the reaction (ET or PT) becoming more thermodynamically favoured. With this in mind, catalysts and reactions can be designed such that changes in pH result in changing the mechanism of PCET between stepwise or concerted PCET.^5,26–29^

The nature of the proton (either solvated or at chemically accessible sites from an acidic bond or organic acids) can be the driver for tuning the thermodynamics of the proton-transfer step in the chemical reactivity. By tuning the p*K*_a_ of organic acids, the rate and mechanism of PCET in small-molecule inorganic complexes can be systematically dialled in.^22,23,30,31^ Further, strong electronic coupling between molecular sites on electrodes has been shown to cause reactivity that fundamentally diverges from that of solution-phase or surface-tethered analogues and can be correlated to the acid/base behaviour of the molecular analogue.^17^ Many recent mechanistic studies involved photo-induced PCET of bio-mimic model systems.^32^ Photo-induced PCET model systems, both molecular and biological, have been used to quantify the influence of proton-transfer driving force and transfer distance on the kinetics of PCET.^33–38^ Because of the complex nature of PCET, it remains important to understand mechanisms and factors that govern electron and proton transfer at electrochemical interfaces of a wide variety of molecular and materials systems.

Synthetically facile molecular model systems are important to form the basis for the study for PCET. Azo dyes possess such facile synthetic procedures that offer rich synthetic versatility and allow for the development of a wide range of model systems in which thermodynamic properties can be systematically tuned. Applications of azo dyes include organic solar cells, molecular photoswitches, protein probes, molecular machines, and non-linear optical components, which encompasses a wide range of applications-driven systems.^39–42^

**Fig. 1:**
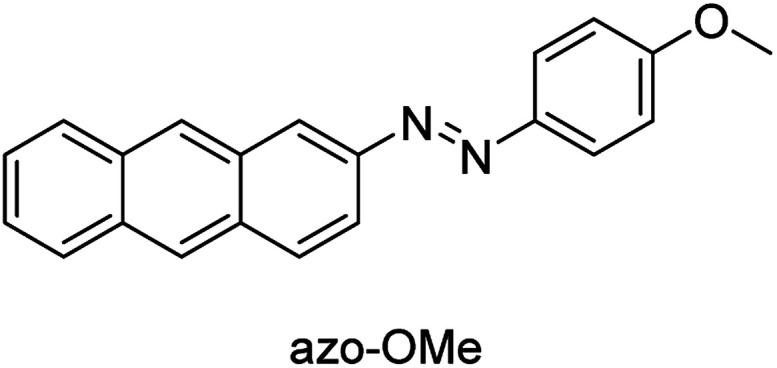
(Anthracen-2-yl)-2-(4-methoxyphenyl)diazene (azo-OMe).

In previous work from our group, we described the design and study of (anthracen-2-yl)-2-(4-methoxyphenyl)diazene (azo-OMe, Fig. 1.) as a model system for the study of photo-induced PCET. Although the photochemistry was not perturbed in the presence of 1 equivalents (eq.) of trifluoroacetic acid (TFA), we found a large positive potential shift in the presence of 1 eq. TFA *versus* 0 eq. TFA.^43^ This work builds upon these initial electrochemical measurements to fully understand the electrochemical PCET reactivity of this anthracene-based azo dye. Towards this end, we have probed the electrochemical reaction of azo-OMe using several different organic acids with acidity constants spanning the p*K*_a_ range of 2.6–23.51 in acetonitrile using the combination of cyclic voltammetry, differential pulse voltammetry, and rotating-disk electrode voltammetry along with a kinetic isotope dependence study. Each of the electrochemical reduction reactions are show to be diffusion controlled at the electrode surface, and are kinetically limited by the electron-transfer step. We show that the PCET mechanism is tuned by the variation of the organic acid p*K*_a_, which serves a proxy (or stand in) for pH in non-aqueous conditions. Using a combination of rotating disk voltammetry and kinetic isotope effect studies, we show that in solutions with a less acidic acid (*i.e.* acids with p*K*_a_ values greater than the p*K*_a_ of azo-OMe), azo-OMe undergoes a concerted 1H^+^/1e^−^ transfer PCET redox reaction. The activation energy of the concerted reaction is less than the sum of the individual proton- and electron-transfer steps. However, in the presence of a highly acidic organic acid (*i.e.* acids with p*K*_a_ values less than the p*K*_a_ of azo-OMe), such as HDMF OTf, a proton transfer occurs prior to the electrochemical electron transfer, indicating that the activation energy of the proton-transfer step is sufficiently decreased to decouple the concerted PCET reaction.

## Results and discussion

### Electrochemical reduction potentials depend upon the p*K*_a_ of added acids

The redox properties of azo-OMe were measured with cyclic voltammetry (CV) and differential pulse voltammetry (DPV). To probe the electrochemical PCET reaction, nine organic acids were used, with a p*K*_a_ range in acetonitrile of 2.6–23.51 (Table S1, ESI†) in order to tune the proton-dependent thermodynamics of the reaction while measuring the electron-transfer reactivity. These data may be used to construct a potential-p*K*_a_ diagram (*vide infra*) that yields a complete ET/PT thermodynamic picture of azo-OMe. A 10 mM stock solution of acid with 0.5 mM azo-OMe in acetonitrile (100 mM TBAPF_6_) was titrated into a solution of 0.5 mM azo-OMe (100 mM TBAPF_6_) at 100 and 500 μL increments (0.19–2.33 eq. of acid). CV was acquired upon each addition of acid and DPV was acquired at 1 and 2 eq. of acid.

**Fig. 2:**
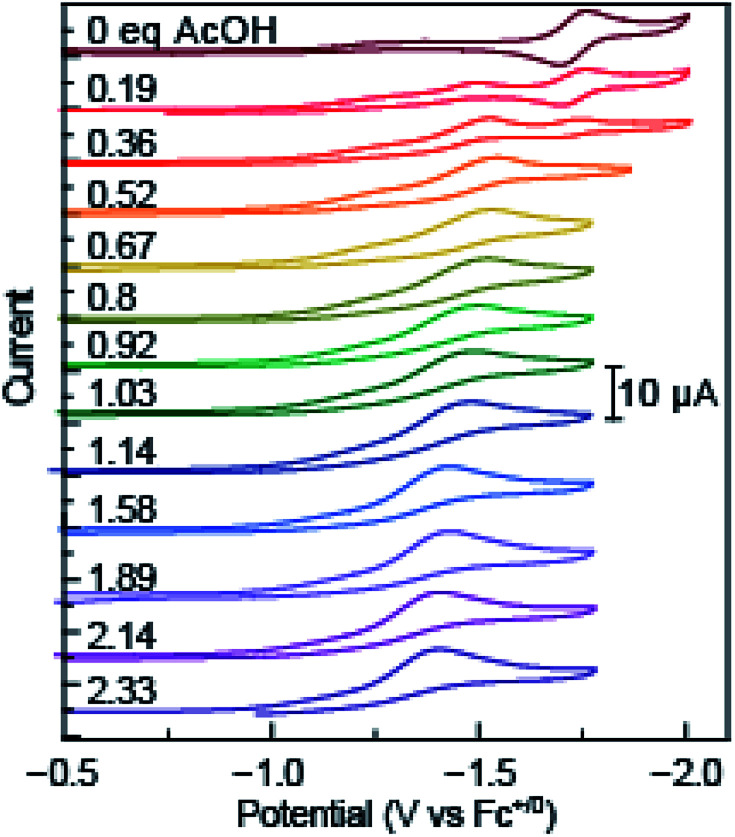
Overlaid cyclic voltammetry scans of azo-OMe acid titration with 0 equivalent to 2.33 equivalent acetic acid (p*K*_a_ (MeCN) = 23.51). Inset: scale of current response in μA.

Consistent with previous results, the reversible reduction peak of azo-OMe in acetonitrile under inert and aprotic conditions was *E*_1/2_ = −1.7 V *vs.* Fc^+/0^ before addition of any acid.^43^ Upon the addition of small amounts (<∼0.5 equivalents) of each acid, the reversible reduction peak disappeared concomitant with the growth of an irreversible reduction peak at more positive potentials. As additional acid was added (>0.5 equivalents), the initial reversible peak continued to disappear and the first irreversible reduction peak also disappeared to yield another, even more positively shifted irreversible reduction peak The irreversible peaks were shifted to positive potentials, meaning that reduction became more facile upon the addition of acid (Fig. 2 and S1–S8, ESI†). As the acid titrations reached the 2 equivalent acid added point (and up to 2.33 equivalents) the reduction peak ceased to significantly shift.

As expected, the Δ*E* of the peak shift depended intimately on the p*K*_a_ of the acid added, a hallmark of proton-coupled electron-transfer reactions. Acids with higher p*K*_a_ values (in acetonitrile) resulted in a smaller positive shift of the reduction potential. For example, addition of acetic acid (p*K*_a_ (MeCN) = 23.51) resulted in only a 300 mV shift from *E*_1/2_ = −1.7 V *vs.* Fc^+/0^ (no added acid) to *E*_1/2_ = −1.38 V *vs.* Fc^+/0^ upon addition of 2 equivalents of acid. Whereas, acids with lower p*K*_a_ values (in acetonitrile) resulted in significantly larger changes in the reduction potential. Specifically, dimethylformamidium trifluoromethanesulfonic acid (HDMF) (p*K*_a_ (MeCN) = 2.6) resulted in a 950 mV shift to *E*_1/2_ = −0.45 V *vs.* Fc^+/0^ upon addition of 2 equivalents of acid. By utilizing organic acids with p*K*_a_ values spanning this range, the thermodynamics of azo-OMe and any reactions that occur using it can be experimentally determined.

### Potential-p*K*_a_ diagram

Potential-p*K*_a_ diagrams have been developed to understand PCET reactions in non-aqueous solutions for which Pourbaix diagrams that are used for aqueous systems cannot be employed. Because solvated protons do not exist in organic solvents the way they do in aqueous media, the pH scale of a Pourbaix diagram must be modified. Instead of pH, the p*K*_a_ of an organic acid can be used as the proxy for the thermodynamic availability of the proton in a potential-p*K*_a_ diagram.^44^ The potential-p*K*_a_ diagram plots the electrochemical reduction or oxidation potential of a process of interest *versus* the p*K*_a_ of a range of added organic acids. These plots show regions in which various electrochemical and protonation states of the analyte, in this case the azo-OMe, exist as a function of proton availability (from an added organic acid).

**Fig. 3:**
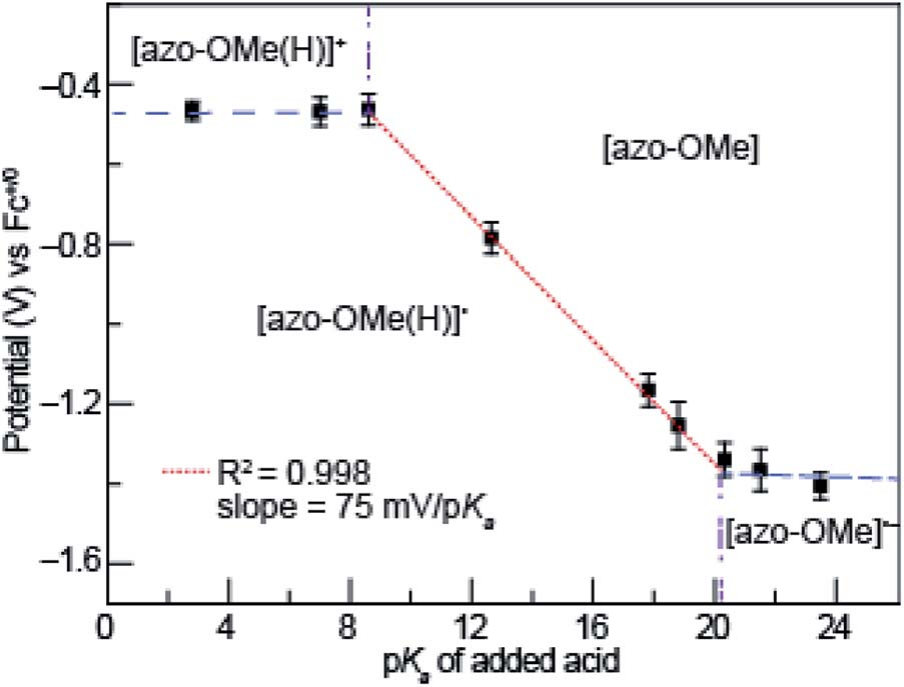
Potential-p*K*_a_ diagram of azo-OMe with 2 eq. of acid.

To construct the potential-p*K*_a_ diagram for azo-OMe, the reduction potential of azo-OMe was measured using cyclic voltammograms that were acquired with 0.5 mM azo-OMe, 2 equivalents of acid, and 100 mM TBAPF_6_ in acetonitrile. Fig. 3 shows the peak potential of azo-OMe (*vs.* Fc^+/0^) plotted *versus* the corresponding p*K*_a_ of each added acid. The potential-p*K*_a_ diagram for azo-OMe reveals one p*K*_a_-dependent and two p*K*_a_-independent regions. One p*K*_a_-independent region occurred when strongly acidic acids (p*K*_a_ 2.6 to 8.6) are used. In this region, the results suggests that azo-OMe is protonated prior to reduction and that in this case, the reduction potential of the protonated azo-OMe dye is measured directly. The other p*K*_a_-independent region occurred when weaker acids (p*K*_a_ 20.35 to 23.51) were used. In this region azo-OMe is likely not significantly protonated prior to or during the reduction event. The acids with p*K*_a_ > 20.35 are not acidic enough to protonate either the reduced or neutral azo-OMe species. A p*K*_a_-dependent region is observed between (p*K*_a_ 2.6 to 8.6). The slope of the p*K*_a_-dependent region (p*K*_a_ 8.6 to 20.35) is 75 mV per decade, which indicates that this process involves 1H^+^/1e^−^ transfer event. The difference from the idealized Nernstian slope (59 mV per decade) is not substantially large and due to homoconjugation of organic acids in acetonitrile.^44^ While the process could be a 2H^+^/2e^−^ process, the current response associated with the azo-OMe PCET reaction does not in fact double when the acid concentration is increased from 1 to 2 equivalents of acid, thereby ruling out that possibility.

The intersection of the p*K*_a_ dependent and independent regions can be used to predict the p*K*_a_ of [azo-OMe(H)]^+^ and [azo-OMe(H)], which are approximately 8.6 and 20.1 respectively. Taken together, lines drawn through each p*K*_a_ dependent (Fig. 3, blue dashed) and independent regions (Fig. 3, red dotted), as well as vertical lines at their intersection (Fig. 3, dot-dashed purple) can be used create the organic-solvent version of the Pourbaix diagram, the potential-p*K*_a_ diagram.

### Rotating disk electrode voltammetry

**Fig. 4:**
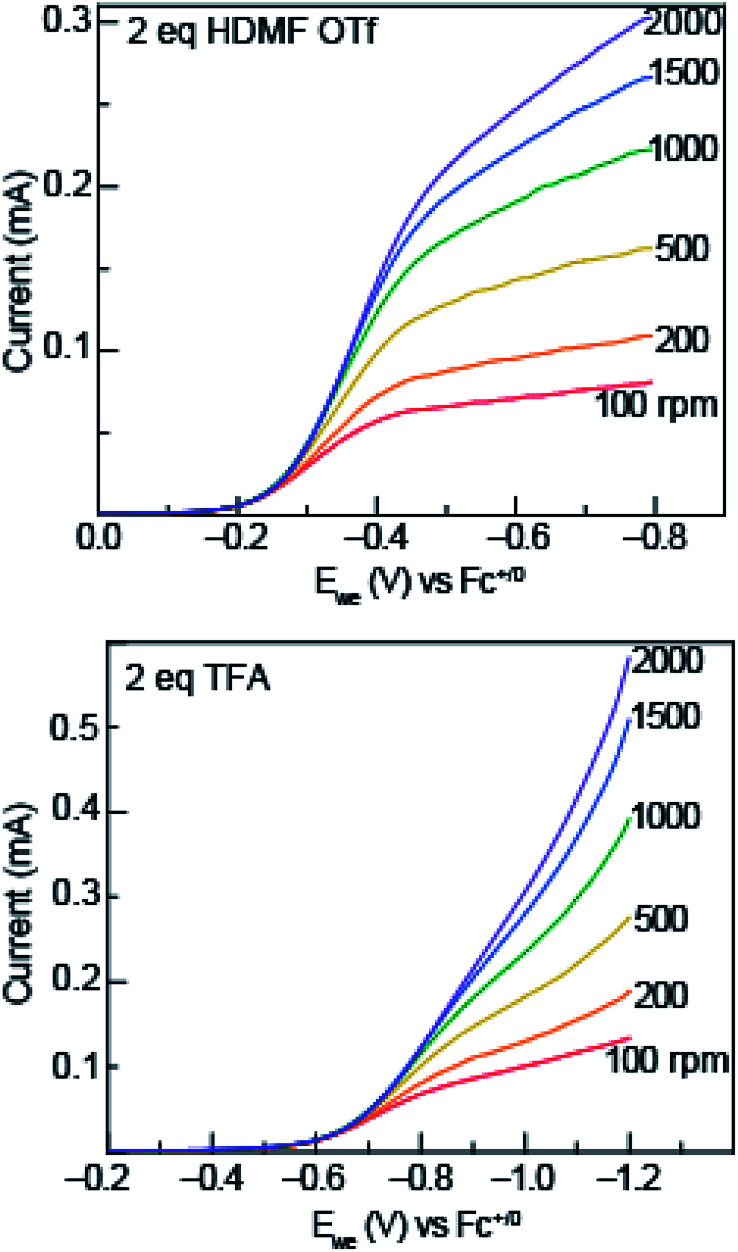
Representative rotating ring disk voltammograms of 0.5 mM azo-OMe with 2 eq. HDMF OTf (top) and TFA (bottom) in 100 mM TBAPF_6_ (MeCN) at rotations rates 100, 200, 500, 1000, 1500, and 2000 rpm and are labelled directly on the plot. *E*_we_ is the potential at the working electrode. Inset: scale of current response in mA.

To garner mechanistic understanding of electrochemical PCET processes, rotating disk electrode (RDE) voltammograms were acquired with solutions of 0.5 mM azo-OMe and 2 eq. of acid (HDMF OTf, or TFA) at various rotation rates (*ω* = 100, 200, 500, 1000, 1500, and 2000 rpm). HDMF OTf and TFA were chosen because they fall into the two relevant p*K*_a_ ranges for PCET reactions with azo-OMe, according to the potential-p*K*_a_ plot. HDMF OTf is highly acidic and protonates azo-OMe even when not reduced, and TFA falls into the p*K*_a_-dependent region and is acidic enough to protonate the reduced azo-OMe species. Fig. 4 shows RDE voltammograms of HDMF OTf (top) and TFA (bottom). For 2 eq. HDMF OTf voltammograms, the current increases as more reducing potentials are applied at a given rpm until a plateau region is reached at approximately −0.4 V *vs.* Fc^+/0^. For the 2 eq. TFA voltammograms, the onset of current is shifted to more reducing potentials (∼−0.6 V *vs.* Fc^+/0^ rather than −0.2 V *vs.* Fc^+/0^ for the HDMF voltammograms). The plateau current was not observed in the RDE plot with 2 eq. of TFA because proton reduction occurs *via* the glassy carbon electrode at −1.2 V *vs.* Fc^+/0^, which begins to dominate the current response. The disk current of azo-OMe with 2 eq. of HDMF OTf and 2 eq. of TFA exhibited Levich behaviour, which is the increase in current response as the rotation rate is increased. The Levich behaviour observed when the rotation rate was increased (Fig. 4), indicates that the reaction is diffusion controlled. RDE voltammograms were used to garner information about the kinetics and mechanism of the PCET reaction of azo-OMe and 2 equivalents of acid through a Koutecký–Levich analysis (*vide infra*).

### Koutecký–Levich and Tafel analysis

**Fig. 5:**
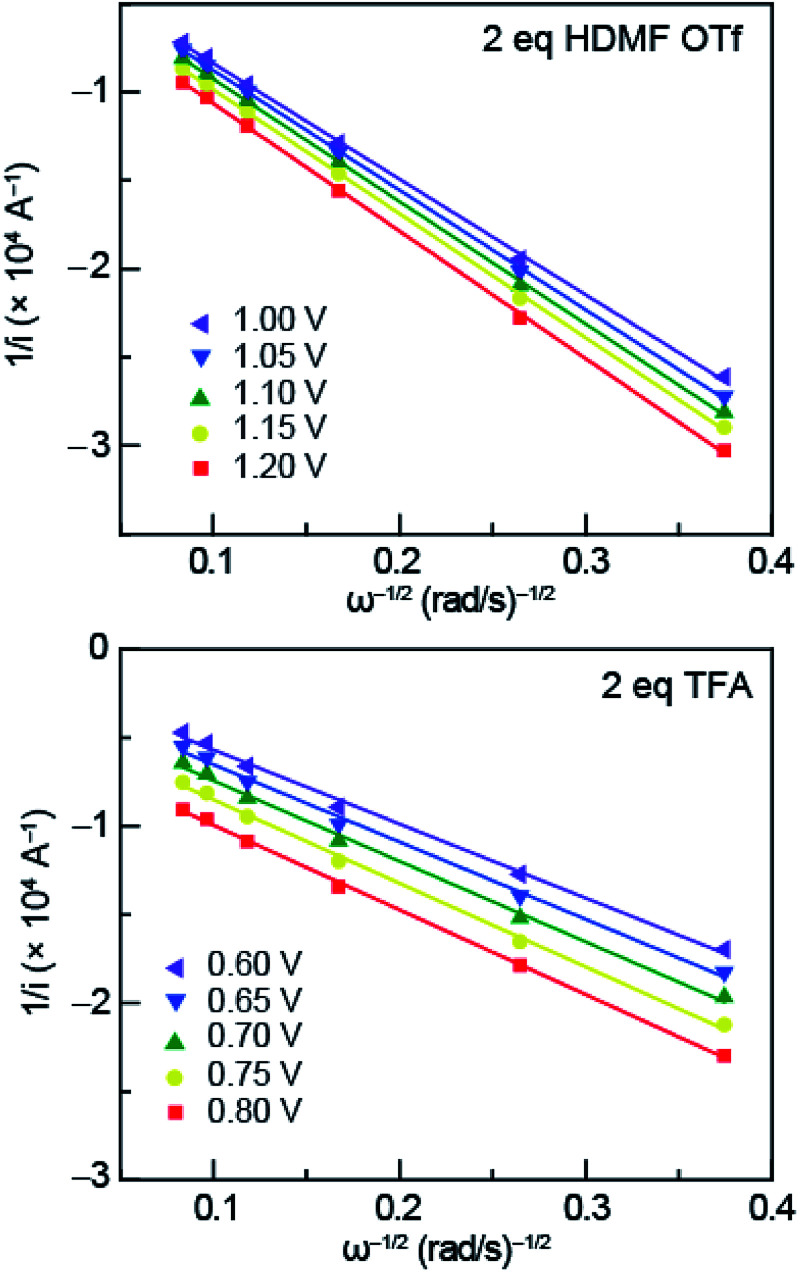
Representative Koutecký–Levich plots of 0.5 mM azo-OMe with 2 eq. HDMF OTf (top) and TFA (bottom) in 100 mM TBAPF_6_ (MeCN) at various overpotentials. The data was plotted using the linear relationship described by the Koutecký–Levich equation (eqn (1)).

The data obtained from RDE experiments were plotted using the Koutecký–Levich (KL) eqn (1)1
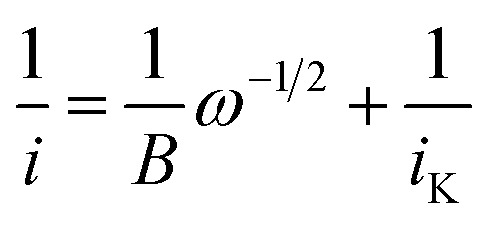
wherein *i* is the measured current (A), *B* is the Levich constant, *ω* is the angular rotation rate (rad s^−1^), and *i*_K_ is the kinetic current. The KL plots of azo-OMe with 2 eq. of HDMF OTf and azo-OMe with 2 eq. TFA are shown in Fig. 5.

The reciprocal of measured currents, obtained from the rotating-disk voltammograms over a range of overpotentials that fall within the plateau region, were plotted *versus* the reciprocal of the square root of rotation rate. The reaction overpotential (*η*) is defined as the difference between the peak potential of azo-OMe^0/−^ (*E*) in the presence of acid and the half potential of azo-OMe^0/−^ (−1.7 V = *E*_eq._) eqn (2).

2 *η* = *E* − *E*_eq._

The KL plots at various overpotentials do not intersect the origin of the *y*-axis, which indicates that the reaction is also kinetically limited by the electron-transfer step. The linear KL plots were extrapolated to yield the *y*-intercept, 1/*i*_K_, where current would be kinetically limited, but not limited under diffusion control. The separation of the kinetically-limited and diffusion-limited processes using KL treatment allows for the determination of heterogeneous electron-transfer rate constants.

**Fig. 6:**
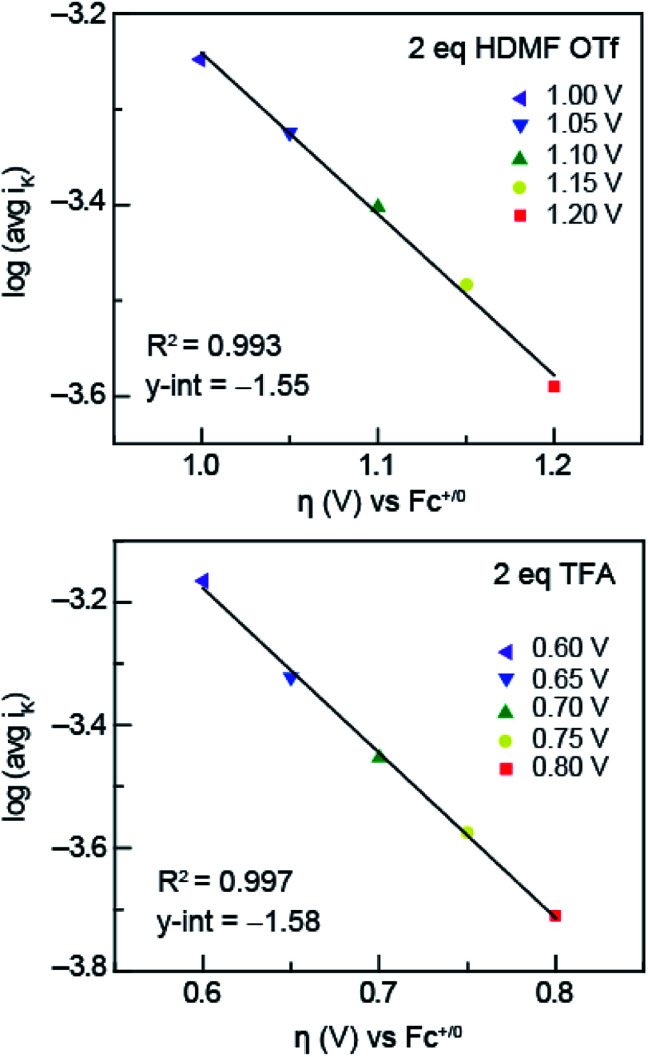
Tafel plots of HDMF OTf (top) and TFA (bottom) used to obtain *k*_s_. The inverse of the KL *y*-intercept and overpotential was plotted using the relationship furnished by the Tafel equation (eqn (3)).

To obtain the exchange current, *i*_0_, log *i*_K_ (*i*_K_ = inverse of the KL *y*-intercept) was plotted *versus* overpotential, shown in Fig. 6, using the Tafel equation, eqn (3),3
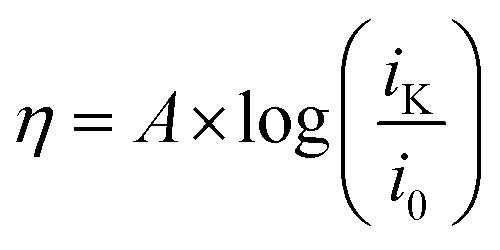
wherein *η* is overpotential, *A* is the Tafel slope, *i*_K_ is the kinetic current, and *i*_0_ is the exchange current. We can use the exchange current to calculate the heterogeneous electron-transfer rate constant, *k*_s_, which was obtained using eqn (4),

4 *i*_0_ = *k*_s_*FAC*

wherein, *F* is Faraday constant, *A* is surface area of the electrode, *C* is the concentration of azo-OMe, and *k*_s_ is the heterogeneous electron-transfer rate constant. The heterogeneous rate constants obtained are summarized in Table 1 for azo-OMe with 2 equivalents HMDF OTf and azo-OMe with 2 equivalents TFA.

**Table 1:** Heterogeneous electron-transfer rate constants (*k*_s_)

Acid	*k*_s_ (cm s^−1^)
HDMF OTf	0.614 ± 0.008
TFA	0.587 ± 0.005
d_1_-TFA	0.245 ± 0.007

In solutions of 2 equivalents HDMF OTf with azo-OMe, the electron transfer is found to be faster (*k*_s_ = 0.614 ± 0.008 cm s^−1^) than the electron transfer than when 2 equivalents of TFA with azo-OMe is used (*k*_s_ = 0.587 ± 0.005 cm s^−1^). This observation can be understood by considering the thermodynamics of proton transfer before the electron transfer is initiated. The p*K*_a_ (MeCN) of HDMF OTf is 2.6, four orders of magnitude more acidic than the azo-OMe, which is estimated to be 8.6 from the potential-p*K*_a_ analysis (Fig. 3). Therefore, azo-OMe is overwhelmingly protonated in solution of HDMF OTf prior to an ET event, which allows for a faster electron transfer. In contrast, the p*K*_a_ of TFA (p*K*_a_ = 12.6 in MeCN) is less than that of azo-OMe, meaning that the azo-OMe is not appreciably protonated before the electron transfer event. However, upon reduction, the azo-OMe will be certainly be protonated (p*K*_a_ [azo-OMe]^−^ = ∼20 in MeCN) because the reduced species is far less acidic than the TFA. Because both the electron and proton transfer must occur in this event, the electron transfer is *k*_s_ = 0.587 ± 0.005 cm s^−1^, slower than the more acidic solutions. With 2 equivalents of TFA, azo-OMe is not protonated prior to electron transfer, but from this analysis, it is not clear whether there is concerted electron–proton transfer (CPET) or stepwise electron, then proton transfer (ETPT). To distinguish between these processes, kinetic isotope experiments were performed.

### Kinetic isotope effect with deuterated TFA

**Fig. 7:**
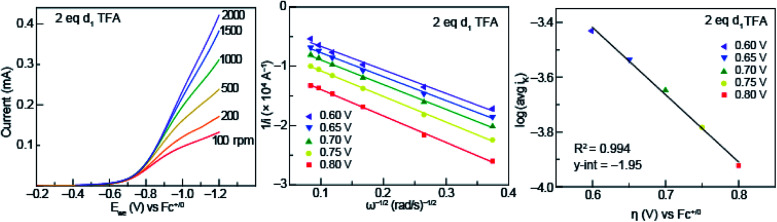
(Left) Representative rotating ring disk voltammograms of 0.5 mM azo-OMe with 2 eq. d_1_ TFA in 100 mM TBAPF_6_ (MeCN) at rotations rates 100, 200, 500, 1000, 1500, and 2000 rpm. *E*_we_ is the potential at the working electrode. Inset: scale of current response in mA. (Center) Representative Koutecký–Levich plots of 0.5 mM azo-OMe with 2 eq. d_1_ TFA in 100 mM TBAPF_6_ (MeCN) at various overpotentials (per eqn (1)). (Right) Tafel plot of d_1_ TFA used to obtain *k*_s_ (per eqn (3)).

To garner information about the proton-coupled electron-transfer mechanism of less acidic acids (acids that fall into the p*K*_a_-dependent range of the potential-p*K*_a_ plot of Fig. 3), RDE experiments were performed with solutions of 0.5 mM azo-OMe and 2 equivalent of deuterated TFA at various rotation rates (Fig. 7, left). In similar fashion to those of the protonated acids, the RDE voltammograms with deuterated TFA also show Levich behavior, which indicates that the deuterated reaction is also under diffusion control.

The data obtained from rotating disk experiments was plotted with Koutecký–Levich and Tafel analysis, shown in Fig. 7 (center and right, respectively). The *y*-intercept of the Koutecký–Levich plots with d_1_-TFA did not intersect the origin, which indicates the electron-transfer step is also kinetically limited for the deuterated reaction. The heterogeneous rate constant obtained from Tafel analysis is shown in Table 1.

A KIE (*k*_s,H_/*k*_s,D_) of 1.8 was found, which is consistent with a concerted proton- and electron-transfer mechanism.^24,32^ Previous reports by Costentin *et al.* indicated an intramolecular electrochemical CPET of a substituted-phenol with a nearby amine group (KIE of 1.8).^45^

Along the p*K*_a_-dependent region of the potential-p*K*_a_ diagram, a PTET mechanism can be excluded due to the difference in p*K*_a_ of [azo-OMe(H)]^+^ (≈8.6 in MeCN) and weak organic acids, such as TFA (Δp*K*_a_ = p*K*_a_(TFA) − p*K*_a_([azo-OMe(H)]^+^) ≈ 4). A protonation prior to an electron-transfer in this regime would be too energetically uphill and a concerted mechanism would bypass that high energy intermediate. An ETPT mechanism can be excluded based on the magnitude of KIE (although small), which is consistent with the involvement of PT in the rate-determining electron transfer step.^46,47^

## Conclusions

To understand the electrochemical PCET reactivity, this work builds upon initial electrochemical measurements of azo-OMe previously reported by our laboratory.^43^ In this work, we have probed the proton-dependence of the electrochemical reaction of azo-OMe using nine organic acids, with acidity constants spanning the p*K*_a_ range of 2.6–23.51 in acetonitrile, using a combination of electrochemical techniques. Our goal was to control the kinetics and mechanism of the PCET reaction at an electrode surface by tuning the thermodynamic (and by extension of transition state theory) the kinetics of the PCET reaction.

The data obtain from cyclic voltammetry experiments was used to construct a potential-p*K*_a_ diagram, which indicated a 1H^+^/1e^−^ PCET process occurrs in the presence of acids with p*K*_a_ values spanning p*K*_a_ 8 to 20. The potential-p*K*_a_ diagram was also used to predict the p*K*_a_ of [azo-OMe(H)]^+^ and [azo-OMe(H)], which are approximately 8.6 and 20.1 respectively.

Rotating disk voltammetry with Koutecký–Levich and Tafel analysis was used to obtain a mechanistic understanding of the PCET process azo-OMe undergoes in the presence of added organic acids. These analyses shows that the PCET reactions are diffusion limited and are kinetically controlled by the electron-transfer step. Due to the p*K*_a_ difference between azo-OMe and HDMF OTf (p*K*_a_ (MeCN) = 2.6), azo-OMe is overwhelmingly protonated in solution of 2 equivalents HDMF OTf prior to an electron transfer event, which allows for a faster electron transfer (*k*_s_ = 0.614 ± 0.008 cm s^−1^). In solution of 2 equivalents TFA (p*K*_a_ (MeCN) = 12.6), *k*_s_ = 0.587 ± 0.005 cm s^−1^, slower than the more acidic solutions. To understand this result, kinetic isotope effect studies were conducted with deuterated TFA to distinguish if the PCET event in the less acidic TFA was concerted (CPET) or stepwise (ETPT). A KIE of 1.8 was obtained, which indicates that the electron and proton are transferred in a concerted step.

Our results demonstrate that azo-based dyes exhibit rich electrochemical PCET chemistry – the mechanism of which can be tuned using the proton-transfer thermodynamics. These results represent some of the first examples using azo-based dyes that examine the PCET mechanism at electrode surfaces in which the proton originates from organic acids introduced into the solution, rather than *via* an intramolecular processes, which have been studied in a limited fashion prior to this.^20^ Ongoing efforts to vary the identity of arene on the anthracene side of the azo bond as well as the R group on the phenyl side are currently underway. Further, work is underway to probe the influence of azo bond protonation on the ultra-fast excited state dynamics of the azo dyes for which the thermodynamic properties reported in this work are essential.

## Experimental

### Materials and synthetic procedures

All reagents were obtained from commercial sources and used as received without further purification, unless otherwise specified. The anthracene-based azo dye, (anthracen-2-yl)-2-(4-methoxyphenyl)diazene (azo-OMe) was synthesized according to literature procedure.^43^

### Cyclic voltammetry and differential pulse voltammetry

All cyclic voltammetry (CV) and differential pulse voltammetry (DPV) measurements were recorded on a CH Instruments potentiostat/galvanostat. CV and DPV were performed using a glassy carbon disk (3 mm diameter) working electrode, a platinum wire counter electrode, and a silver wire quasi reference electrode. The glassy carbon working electrode was polished with 0.05 μM alumina powder/water slurry, rinsed with water and acetone, and allowed to air dry prior to each experiment. Tetrabutylammonium hexafluorophospate (TBAPF_6_) was recrystallized with hot ethanol three times, dried under vacuum, and used as the supporting electrolyte. Acetonitrile was pre-dried with 4 Å molecular sieves, distilled over calcium hydride, and stored over 4 Å molecular sieves prior to use. Ferrocene was used as an internal standard and all voltammograms were reported *vs.* Fc^+/0^ couple. The scan rate used for CV was 100 mV s^−1^ unless otherwise specified. All samples were sparged with N_2_ for 10 minutes before CV and DPV and kept under a N_2_ blanket during acquisition.

### Rotating disk voltammetry

All rotating disk experiments were acquired with a Bio-Logic SP-300 bipotentiostat/galvanostat and a Pine MSR rotator. A glassy carbon fixed-disk PEEK shroud working electrode and a platinum mesh counter electrode were used for RDE experiments. The glassy carbon disk was polished with 0.05 μM alumina powder/water slurry, rinsed with water and acetone, and allowed to air dry prior to each experiment. Acetonitrile was purified from a Pure Process Technology free standing solvent purification system. The scan rate used for RDE was 20 mV s^−1^ with rotation rates (*ω*) of 100, 200, 500, 1000, 1500, and 2000 rpm. All solutions were sparged with N_2_ for 10 minutes before RDE and were blanketed with N_2_ during acquisition.

## Conflicts of interest

There are no conflicts to declare.

## Supplementary Material

RA-010-D0RA01643H-s001
