# Supplementary material for: Electrochemical proton-coupled electron transfer of an anthracene-based azo dye
Source: RSC Adv. 2020 Apr 14;10(25):14804–11. doi: 10.1039/d0ra01643h (PMC9052096; doi:10.1039/d0ra01643h)
Supplement: RA-010-D0RA01643H-s001 [file RA-010-D0RA01643H-s001.pdf]

Electronic Supplementary Material (ESI) for RSC Advances.
This journal is © The Royal Society of Chemistry 2020

Investigation of Electrochemical Proton-Coupled Electron Transfer of Anthracene-based Azo dye

Amanda N. Oldacre[a,b] and Elizabeth R. Young[b*]

a. Department of Chemistry, St. Lawrence University, Canton, NY, 13617, USA

b. Department of Chemistry, Lehigh University, Bethlehem, Pennsylvania 18015, USA.

Table of Contents

Organic Acids and corresponding pK_a values 2

Cyclic Voltammograms of azo-OMe Acid Titration 2

Koutecký-Levich Tables 6

Organic Acids and corresponding pK_a values

Table S1. Acids used and pK_a in MeCN[1]

Acid	pK_a (MeCN)
dimethylformamidium trifluoromethanesulfonic acid (HDMF)	2.6
4-cyanoanilinium tetrafluoroborate (4CyAn)	7
p-toluenesulfonic acid (*p*TSA)	8.6
trifluoroacetic acid (TFA)	12.65
Pentabromophenol (PBP)	17.83
triethylammonium tetrafluoroborate ($HNEt_3$)	18.82
2,4,6-tribromophenol (Br_3PhOH)	20.35
benzoic acid (BA)	21.51
acetic acid (AcOH)	23.51

Cyclic Voltammograms of azo-OMe Acid Titration

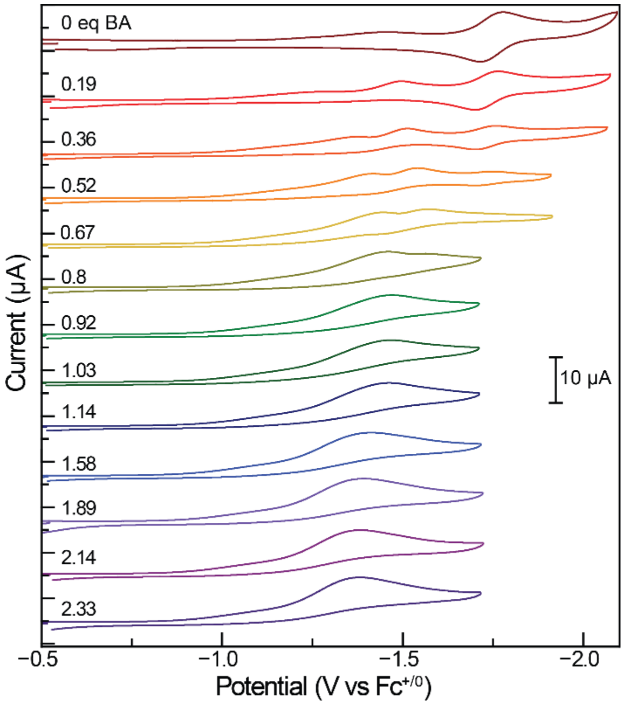

Figure S1. Azo-OMe acid titration with 0 eq to 2.33 eq BA (pK_a (MeCN) = 21.51).

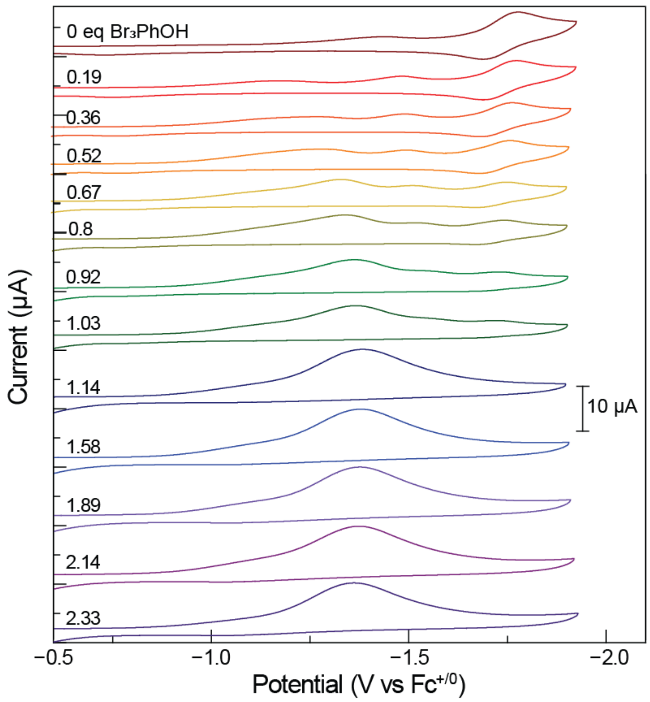

Figure S2. Azo-OMe acid titration with 0 eq to 2.33 eq Br_3PhOH (pK_a (MeCN) = 20.35).

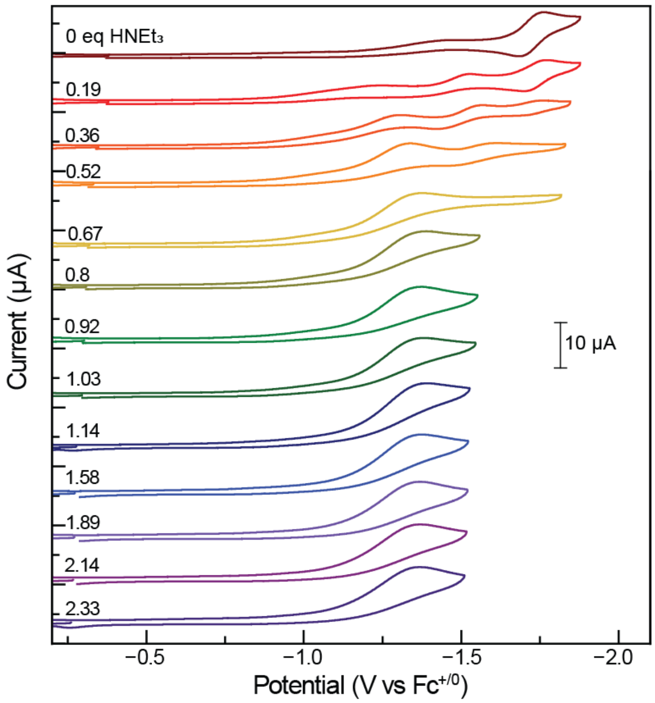

Figure S3. Azo-OMe acid titration with 0 eq to 2.33 eq $HNEt_3$ (pK_a (MeCN) = 18.82).

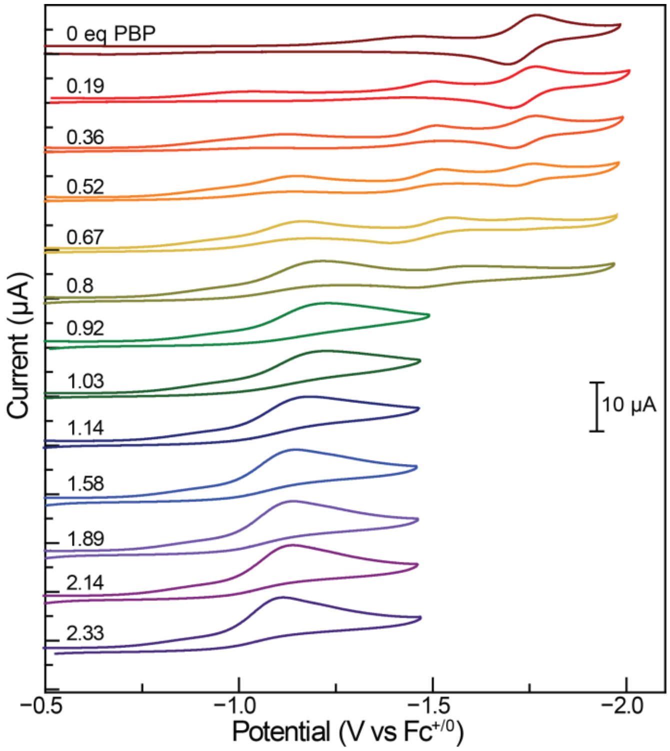

Figure S4. Azo-OMe acid titration with 0 eq to 2.33 eq PBP (pK_a (MeCN) = 17.83).

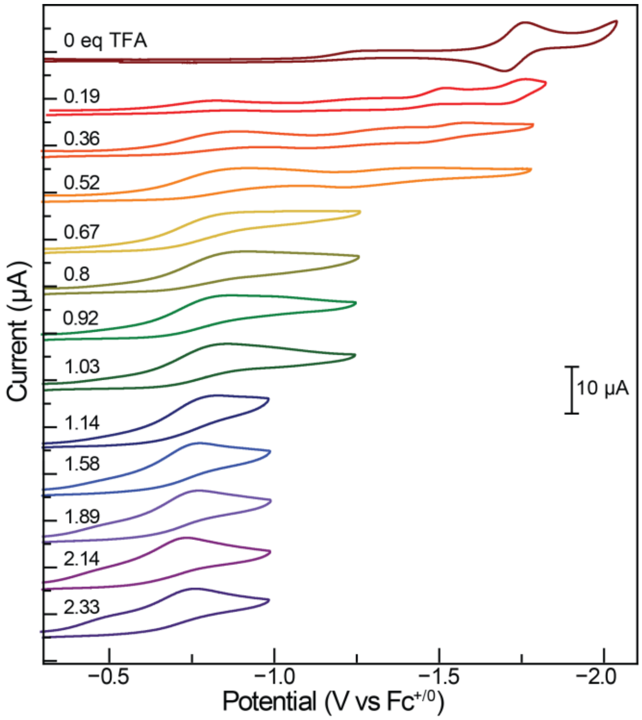

Figure S5. Azo-OMe acid titration with 0 eq to 2.33 eq TFA (pK_a (MeCN) = 12.65).

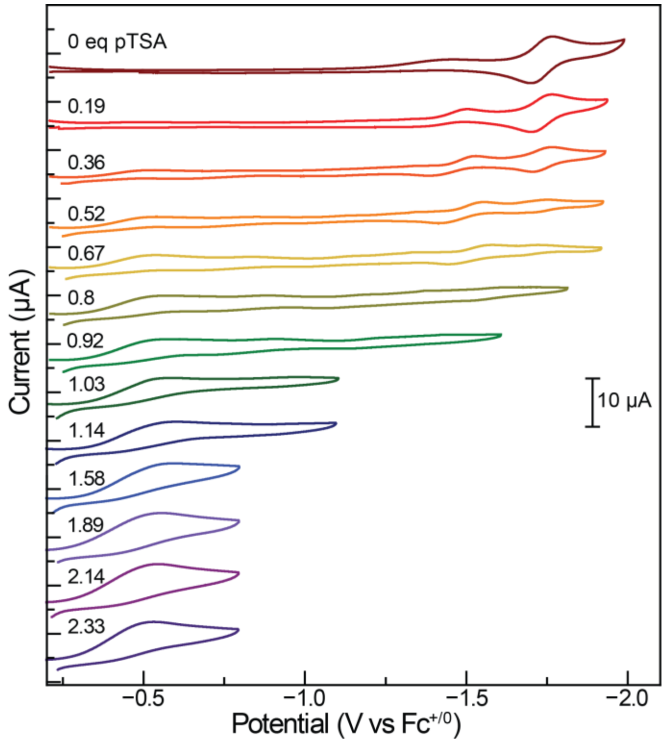

Figure S6. Azo-OMe acid titration with 0 eq to 2.33 eq pTSA (pK_a (MeCN) = 8.6).

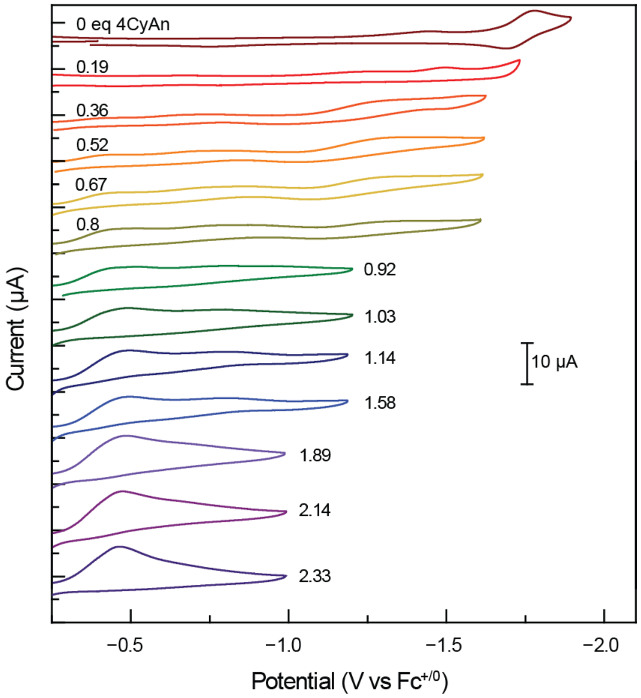

Figure S7. Azo-OMe acid titration with 0 eq to 2.33 eq 4CyAn (pK_a (MeCN) = 7).

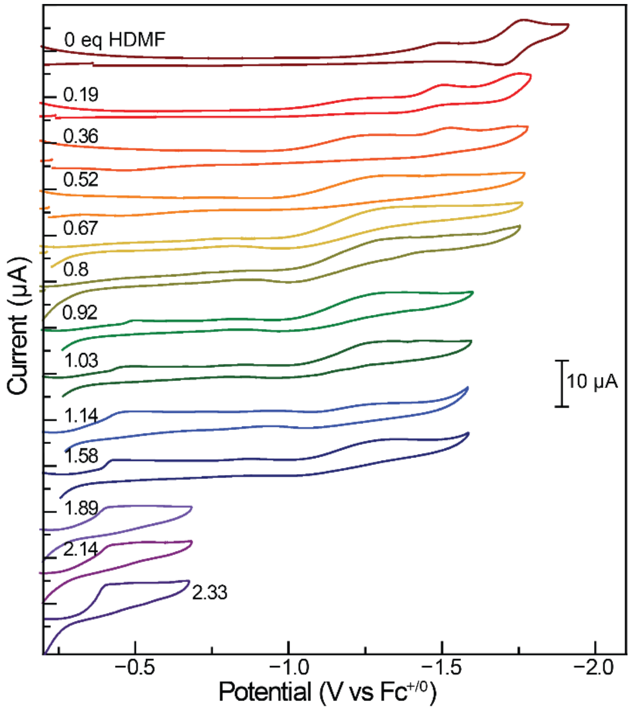

Figure S8. Azo-OMe acid titration with 0 eq to 2.33 eq HDMF (pK_a (MeCN) = 2.6).

Koutecký-Levich Tables

Table S1. Representative table of y-intercepts of azo-OMe and 2 equivalents HDMF OTf

Overpotential (V)	y-intercept	R^2
1.00	−1819	0.999
1.05	−1980	0.999
1.10	−2322	0.999
1.15	−2830	0.999
1.20	−3421	0.999

Table S2. Representative table of y-intercepts of azo-OMe and 2 equivalents TFA

Overpotential (V)	y-intercept	R^2
0.60	−1470	0.995
0.65	−2120	0.995
0.70	−2846	0.996
0.75	−3745	0.997
0.80	−5124	0.999

Table S3. Representative table of y-intercepts of azo-OMe and 2 equivalents deuterated TFA

Overpotential (V)	y-intercept	R^2
0.60	−2685	0.989
0.65	−3678	0.996
0.70	−4769	0.997
0.75	−6457	0.999
0.80	−9443	0.998

References

(1) McCarthy, B. D.; Dempsey, J. L. Decoding Proton-Coupled Electron Transfer with Potential-p*Ka* Diagrams. *Inorg. Chem.* **2017**, *56*, 1225–1231.
